# Nursing and planetary health: A discussion article

**DOI:** 10.1177/17455057241311955

**Published:** 2025-03-12

**Authors:** Salima Meherali, Saba Nisa, Yared Asmare Aynalem, Zohra S Lassi

**Affiliations:** 1 Faculty of Nursing, University of Alberta, Edmonton, AB, Canada; 2 School of Public Health, Faculty of Health and Medical Sciences, The University of Adelaide, Adelaide, SA, Australia

**Keywords:** planetary health, nursing, education, global health

## Abstract

This discussion article discusses the integration of planetary health into nursing practice and education, highlighting the transformative potential of this approach in improving global health outcomes. Planetary health emphasizes the interdependence between human health and the health of our planet’s ecosystems, advocating for a sustainable approach to healthcare. This article explores how nursing practice can incorporate planetary health principles to address environmental determinants of health and promote sustainable practices. It also discusses the role of nursing education in preparing future practitioners to understand and act on the links between environmental sustainability and health. By aligning nursing education with planetary health objectives and fostering leadership in this area, the nursing profession can contribute significantly to addressing global health challenges, advocating for systemic changes, and implementing practices that protect both human and environmental health.

## Introduction

The seventeen sustainable development goals (SDGs) established by the United Nations form a comprehensive framework addressing many global challenges, including poverty, inequality, climate change, environmental degradation, peace, and justice for all.^1,2^ Aligned with these objectives is the concept of planetary health, emphasizing the intricate interdependence between human health and the planet’s well-being.^3,4^ In an era marked by unprecedented global challenges, the intersection of SDGs and planetary health emerges as a transformative nexus, breaking conventional disciplinary boundaries.^5^ The imperative to confront climate change, biodiversity degradation, and health inequities demands a profound exploration of the intricate relationships within these frameworks. As the global community grapples with the compounding impacts of environmental degradation on human health, recognizing the interdependence of SDGs and planetary health becomes paramount for informing global policy decisions, research initiatives, and practical interventions.^6–8^

As frontline healthcare providers, nurses are uniquely positioned to contribute significantly to achieving SDGs and promoting planetary health.^9,10^ This article contributes significantly to nursing knowledge by filling a crucial gap and comprehensively exploring the interconnected relationships between these transformative frameworks within nursing practice.^11^ Traditionally centered on individual and community health within healthcare settings, nursing knowledge now expands to address the converging challenges of SDGs and planetary health, emphasizing the intricate connections between environmental sustainability, human health, and well-being.^12,13^ Furthermore, the article offers actionable insights and strategic opportunities for nurses to engage in integrated and collaborative solutions, guiding the intricate landscape of interconnected global well-being.^14^ Emphasizing that sustainable development is inseparable from planetary health underscores nursing professionals’ responsibility to advocate for environmentally sustainable and socially equitable practices.^3,15^ The article contributes to shaping a more comprehensive and inclusive nursing knowledge that reflects the complex realities of the contemporary global health context.^16,17^ Through this exploration, the article is a valuable contribution to nursing, fostering a deeper understanding of the discipline’s role in addressing global challenges.

## Discussion

### The paradigm shift: integrating planetary health into the global well-being

The discourses surrounding global well-being have undergone a profound transformation in recent decades. The introduction of “One Health”—a concept first used by Calvin Schwabe in 1964 to encompass the interconnection of human and animal health and codified in the Manhattan Principles in 2004—enabled a joint framework for addressing zoonotic diseases to be supported by several international organizations and has since underpinned the World Health Organization (WHO)’s approach to integrated modern health promotion.^18^ WHO defines health as “a state of complete physical, social, and mental well-being, and not merely the absence of disease or infirmity, and also recognizes the intricate relationship between human well-being and the health of the planet.^19–21^ Moreover, the report of The Rockefeller Foundation–Lancet Commission on Planetary Health^22^ introduced planetary health as the interconnectedness of human health and the Earth’s natural systems, emphasizing the importance of addressing the political, economic, and social factors that influence both. This holistic approach underscores the need for a comprehensive examination of the complex web linking the planet’s health to the overall well-being of individuals and communities.^3,23^

The severity of health-related outcomes stemming from the interconnectedness of human activities and the natural environment has catalyzed the formation of practitioner groups dedicated to promoting climate action within healthcare.^24,25^ Prominent initiatives addressing the intersection of health and the environment include the Global Green and Healthy Hospitals, the Center for Climate Change and Health, the Alliance of Nurses for Healthy Environments, and the Canadian Association of Nurses for the Environment. These organizations symbolize an escalating awareness within the healthcare sector of the urgent need to confront challenges posed by climate change directly to Health.^26–28^

### Planetary health and sustainable development goals

Planetary Health and the SDGs are closely intertwined, addressing Earth’s and its inhabitants’ well-being. Concurrently, the SDGs, a set of 17 global objectives established by the United Nations, aim to tackle social, economic, and environmental challenges to create a more sustainable world by 2030.^29,30^ Each SDG reflects a specific aspect of this interconnected relationship. Goal 3, which is focused on good health and well-being, aligns directly with planetary health by emphasizing the need for healthy lives and well-being across all age groups.^31^ Goal 6, clean water and sanitation, recognizes sustainable water management’s vital role in supporting human health and ecosystem well-being.^32,33^ Goal 13, climate action, acknowledges the urgent connection between planetary health and climate change, advocating for measures to combat its impacts.^9^ Goals 14 (Life Below Water) and 15 (Life on Land) emphasize conserving marine and terrestrial ecosystems, which are crucial for planetary health and human well-being.^34,35^ Goals 2 (Zero Hunger) and 7 (Affordable and Clean Energy) address issues related to access to nutritious food, sustainable agriculture, and clean energy sources, impacting both human and planetary health.^36^ Goal 11, Sustainable Cities and Communities recognizes the impact of urbanization on human health and the environment, striving for inclusive, safe, resilient, and sustainable cities.^37^ Goal 17, Partnerships for the Goals, underscores the necessity of collaboration, acknowledging that addressing complex challenges in planetary health requires coordinated efforts across sectors and countries.^38^

Nursing contributions to planetary health are essential for interdisciplinary efforts, addressing human causes and health implications of environmental degradation. Nurses are uniquely positioned to lead the advancement of planetary health science, aligning with ethical imperatives to advocate for policies that reduce environmental risks and promote health.^39–41^ The American Nurses Association calls upon nurses to integrate climate science into education, research, and practice, advocating for evidence-based actions and policies. Moreover, nurses create community partnerships to promote health and influence policies and legislation, and they are called upon to integrate the science of climate and health into nursing education, research, and practice.^39,42^ The Center for Planetary Health and Environmental Justice (School of Nursing, University of Minnesota) emphasizes the importance of nurses’ involvement in creating healing opportunities for planetary health and advocates for transforming nursing curricula to support planetary health.^43^

Despite commendable progress, especially in nursing, the healthcare sector must respond more proactively to the climate change crisis.^44^ Despite the formation of advocacy groups and increased awareness, systemic changes within healthcare systems have been gradual. Urgency demands a swift and comprehensive integration of climate-conscious practices and policies across the entire healthcare spectrum, aligning with the principles of planetary health for a sustainable future.

### Planetary health and nursing

Nurses are responsible for promoting health, preventing illness, injury, and disease, and protecting the health and the environment.^45^ A shift from “global nursing,” which involves nurses’ role in global health efforts with a focus on social determinants of health and cultural diversity, to “planetary nursing,” which includes broader possibilities for engagement in planetary health initiatives, is needed.^46^ This shift to planetary nursing allows nurses to effectively engage in global health initiatives, such as the United Nations 2030 Agenda for Sustainable Development. Planetary Health incorporates issues beyond the traditional scope of nursing practice, including climate change, biodiversity loss, and changing ecosystems, including increases in vector-borne diseases and epidemics.^45,46^ Furthermore, the recent Future of Nursing 2020–2030 report delves into the intersection of environmental health, racism, discrimination, planetary disasters, and their nursing implications.^22^ The connection between the environment, health, and individuals is integral, and in many theories, nurses are portrayed as change agents for the environment. However, how nurses should enact this role is often unclear. Florence Nightingale (1820–1910) was a pioneering nurse and social reformer widely regarded as the founder of modern nursing. She provides a concrete outline of the nurse’s responsibility to preserve a healthy environment. Despite being rooted in a local conceptualization, Nightingale establishes a well-defined connection between the nurse, the individual, health, and the physical environment—highlighting that a healthy physical environment is fundamentally linked to the healing process. Moving beyond Nightingale, many theorists fail to provide explicit directions on how nurses should function as change agents in the environment, leaving the guidance somewhat unclear.

Despite this lack of specificity, it is evident that nurses consistently prioritize individual health and preferences as their guiding principles. These works present a limited perspective on the nurse-environment relationship, focusing primarily on promoting individual health rather than addressing broader societal concerns. According to a review, 548 articles related to nursing appeared in journals related to climate change and environmental health spanning 1995–2015. The breakdown shows 55 publications between 1995 and 2000, 96 between 2001 and 2005, 170 between 2006 and 2010, and 227 between 2011 and 2018.^47,48^ The rise from 55 articles in 1995–2000 to 227 in 2011–2018 reflects a heightened focus on addressing climate-related health issues within the nursing profession. Given that the scope of nursing practice is continually evolving, planetary health must become a focus for nursing education.

### The call to action

Integrating planetary health into nursing education, policies, and practices must be prioritized as it is essential for preparing future nurses to address the complex interplay between human health and environmental sustainability. The integration of planetary health into nursing curricula is still emerging globally. While some nursing programs are beginning to incorporate aspects of planetary health, the University of Minnesota School of Nursing, established in 1909, emphasizes holistic care and integrates the planetary health education framework to prepare students for health challenges while aligning with SDGs.^49^ The Canadian Association of Schools of Nursing is increasingly recognizing the importance of environmental health, which aligns with global calls for incorporating planetary health into education.^49^ This approach can be made universal; however, several barriers hinder the integration of planetary health into nursing education. The existing nursing curricula are often overloaded, making it difficult to add new content without compromising essential principles. In addition, many faculty members lack training in planetary health, which can impact the quality of teaching. Furthermore, institutional resistance to innovative approaches can impede efforts to update educational frameworks effectively. Furthermore, empowering future nurses to engage in advocacy and promotion of planetary health can be challenging and nurse educators are tasked with ensuring students receive appropriate information on planetary health that can be applied to practice and used to advance health policy on various levels.

### Implications

By embracing planetary health in nursing, we can contribute significantly to achieving the SGDs, fostering a holistic understanding of curative but also preventive and sustainable health for the well-being of current and future generations. Nurses are also encouraged to demonstrate a strong commitment to addressing climate change through various avenues such as research, education, advocacy, and nursing practice. Specifically, contributions focusing on care decisions related to climate-impacted health conditions, the effects of climate change on healthcare delivery and access, sustainable healthcare practices, poverty reduction strategies, community adaptation, and resilience to climate change are sought after. It is recommended to have a comprehensive approach that includes updating nursing curricula to encompass planetary health concepts, emphasizing the interconnectedness of ecosystems and human health. Kalogirou et al.^27^ also recommend adopting planetary health as a theoretical foundation in nursing education, research, and practice. Furthermore, professional development programs should be designed to equip nurses with the skills necessary to address environmental determinants of health and advocate for sustainable healthcare practices. Moreover, research priorities must shift toward planetary health issues, emphasizing the impact of environmental degradation on health.

## Conclusion

This article explores the crucial link between the SDGs and planetary health, emphasizing the transformative role in addressing global challenges. It highlights nurses’ unique contribution amid environmental concerns and advocates for a paradigm shift in nursing education to integrate planetary health principles. Recommendations include adopting these principles, engaging in interdisciplinary care, and promoting sustainable practices. The implications extend to educational restructuring, interdisciplinary collaboration, and policy advocacy, positioning nurses as critical players in shaping a resilient global future. This involvement marks a transformative milestone, establishing nursing as a crucial player in promoting sustainability, resilience, and equity, enriching nursing knowledge for a more comprehensive and inclusive approach to practice.

## Declarations

**Ethical approval and consent to participate:** This study does not require ethical consent because it does not include any human or animal, instead literature review and opinions.

**Availability of data and materials:** Publicly open access.
